# Hypoxia Modulates Platelet Purinergic Signalling Pathways

**DOI:** 10.1055/s-0039-3400305

**Published:** 2019-12-13

**Authors:** Gordon G. Paterson, Jason M. Young, Joseph A. Willson, Christopher J. Graham, Rebecca C. Dru, Eleanor W. Lee, Greig S. Torpey, Sarah R. Walmsley, Melissa V. Chan, Timothy D. Warner, John Kenneth Baillie, Alfred Arthur Roger Thompson

**Affiliations:** 1 APEX (Altitude Physiology Expeditions), Edinburgh, United Kingdom; 2 Edinburgh Medical School, University of Edinburgh, Edinburgh, United Kingdom; 3 University of Edinburgh Centre for Inflammation Research, The Queen's Medical Research Institute, University of Edinburgh, Edinburgh, United Kingdom; 4 Centre for Immunobiology, Blizard Institute, Barts and The London School of Medicine and Dentistry, Queen Mary University of London, London, United Kingdom; 5 Division of Genetics and Genomics, The Roslin Institute, University of Edinburgh, Edinburgh, United Kingdom; 6 Department of Anaesthesia, Critical Care and Pain Medicine, Royal Infirmary of Edinburgh, NHS Lothian, Edinburgh, United Kingdom; 7 Department of Infection, Immunity and Cardiovascular Disease, University of Sheffield, Sheffield, United Kingdom

**Keywords:** hypoxia, platelet physiology, anti-platelet agents, ADP receptors, high altitude

## Abstract

**Background** Hypoxia resulting from ascent to high-altitude or pathological states at sea level is known to increase platelet reactivity. Previous work from our group has suggested that this may be adenosine diphosphate (ADP)-specific. Given the clinical importance of drugs targeting ADP pathways, research into the impact of hypoxia on platelet ADP pathways is highly important.

**Methods** Optimul aggregometry was performed on plasma from 29 lowland residents ascending to 4,700 m, allowing systematic assessment of platelet reactivity in response to several platelet agonists. Aggregometry was also performed in response to ADP in the presence of inhibitors of the two main ADP receptors, P2Y _1_ and P2Y _12_ (MRS2500 and cangrelor, respectively). Phosphorylation of vasodilator-stimulated phosphoprotein (VASP), a key determinant of platelet aggregation, was analysed using the VASPFix assay.

**Results** Hypobaric hypoxia significantly reduced the ability of a fixed concentration of cangrelor to inhibit ADP-induced aggregation and increased basal VASP phosphorylation. However, in the absence of P2Y receptor inhibitors, we did not find evidence of increased platelet sensitivity to any of the agonists tested and found reduced sensitivity to thrombin receptor-activating peptide-6 amide.

**Conclusion** Our results provide evidence of increased P2Y _1_ receptor activity at high altitude and suggest down-regulation of the P2Y _12_ pathway through increased VASP phosphorylation. These changes in ADP pathway activity are of potential therapeutic significance to high-altitude sojourners and hypoxic sea level patients prescribed platelet inhibitors and warrant further investigation.

## Introduction

Acute hypobaric hypoxia, such as that induced by ascent to high altitude, has long been considered to produce a thrombogenic phenotype. 1 Consistent with this, epidemiological studies report a markedly increased risk of strokes at high altitude: up to 30 times that at sea level. 2 Furthermore, these events are reported to occur in younger patients with fewer cardiovascular risk factors. 3 Although some studies examining the effect of acute hypoxia on coagulation have reported minimal changes in coagulation, they were investigating the ‘economy class syndrome’ and thus exposure to hypoxia was brief. 4 5 On the contrary, studies examining a longer exposure to hypoxia have demonstrated a hypercoagulable state. 6 7 8

Platelets are key to haemostasis 9 10 and appear to have an important role in the hypercoagulable state induced by hypoxia: expression of soluble P-selectin, an in vivo platelet activation marker, was shown to be increased 2.5-fold after ascent to high altitude. 11 Furthermore, hypoxia has recently been shown to significantly alter the platelet proteome, including up-regulation of calpain small subunit 1. 12 Calpains are calcium-dependent proteases involved in various physiological processes including platelet activation. 13 The same study also found increased intracellular calcium concentration in hypoxic platelets 12 which is a common downstream effect of platelet activation by several agonists, including adenosine diphosphate (ADP). 10

Recent work from our group has demonstrated a hypercoagulable state in 63 subjects participating in a controlled, non-exertional sojourn to 5,300 m. 6 Multiplate aggregation assays using fixed doses of several platelet activators at this altitude suggested that hypoxia-induced platelet hyper-reactivity is specific to ADP as responses to thrombin receptor-activating peptide (TRAP) and collagen were not altered. 6 It is known that ADP acts on two pro-aggregatory pathways, via P2Y _1_ and P2Y _12_ receptors, 14 and in light of our previous findings we aimed to further investigate these pathways. P2Y _12_ inhibitors are widely used anti-platelet medications, and effects of hypoxia on their efficacy are of high clinical relevance. This is particularly important given the increasing number of older sojourners to high altitude, who may have cardiovascular co-morbidities. 15

Our study is the first to our knowledge to investigate the effect of hypoxia on the major platelet activation pathways with full concentration–response curves. Optimul aggregometry is a recently developed assay which applies the principles of light-transmission aggregometry to a 96-well format, with platelet agonists lyophilised in each well. 16 This assay requires significantly less plasma volume than traditional aggregometry, thus facilitating systematic assessment of platelet aggregation in response to several agonists. Ex vivo use of ADP receptor inhibitors also permitted further detailed study of the purinergic pathways in a group of healthy, lowland volunteers ascending to 4,700 m.

## Methods

Data were collected from 29 participants before and during the APEX 5 Expedition. All participants had no known cardiovascular or respiratory conditions and no pre-existing coagulopathy. Participants were asked to refrain from alcohol and anti-platelet medications during the week prior to sampling. This study was approved by the ACCORD Research Ethics Committee (17-HV-030) and all participants gave informed consent as per the Declaration of Helsinki. Reagents were supplied by Sigma-Aldrich (Irvine, United Kingdom) unless otherwise specified.

### Ascent Profile and Sample Collection

**Fig. 1:**
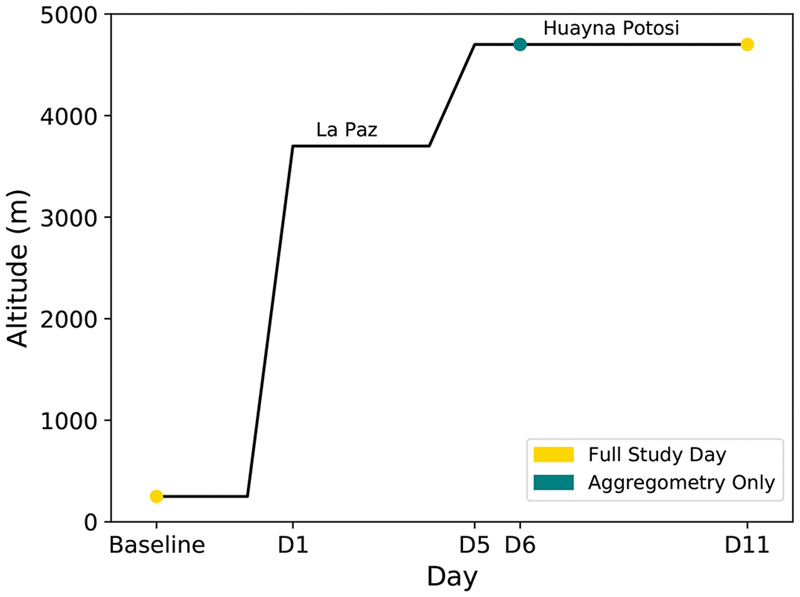
Ascent profile. Baseline testing was performed in April 2017, 2 months prior to the expedition. Subjects landed in La Paz (3,700 m) where they spent four nights before ascending to Huayna Potosi Base Camp (4,700 m) by bus on day 5 where they stayed for the remainder of the study. Optimul aggregometry, VASPFix and full blood count samples were collected at baseline and on day 11. On day 6, only optimul aggregometry was performed.

Participants were resident at < 250 m above sea level and had not travelled to high altitude (> 2,500 m) in the 2 months prior to the study. The ascent profile and study timeline are summarised in Fig. 1 . We chose to perform aggregometry assays on day 6 (one day following ascent to 4,700 m to examine effects of sub-acute hypoxia) and day 11 (the time point we had previously observed changes in Multiplate aggregation with ADP 6 ). Venepuncture was performed using 21G needles (Williams Medical, Rhymney, United Kingdom) into citrated tubes. Platelet-rich plasma (PRP) was prepared by centrifugation of whole blood at 175 × *g* for 15 minutes (EBA 280, Hettich, Tuttlingen, Germany). Platelet-poor plasma (PPP) was generated by further centrifugation at 3,624 × *g* for 2 minutes. Peripheral oxygen saturation (SpO _2_ ) was measured using a pulse oximeter (SM-100, Santamedical, Tustin, United States) at baseline and every day of the expedition.

### Optimul Aggregometry

Modified optimul aggregometry plates were prepared as previously described by Chan and Warner. 16 In brief, pre-diluted platelet agonists were added to individual wells of a gelatin-coated 96-well plate and the plates were lyophilised. Plates were vacuum-sealed and protected from light before use. Each 96-well plate contained lyophilised concentration ranges of arachidonic acid (0.03–1 mM), ADP (0.005–40 µM), collagen (0.01–40 µg/mL), epinephrine (0.0004–10 µM), TRAP-6 amide (0.03–40 µM), and U46619 (thromboxane mimetic, 0.005–40µM).

PRP was aliquoted and incubated for 30 minutes (at 37°C) alone or in the presence of the P2Y _1_ inhibitor MRS2500 (1 µM, Tocris Bioscience, Bristol, United Kingdom) or the P2Y _12_ inhibitor cangrelor (100 nM, The Medicines Company, New Jersey, United States). After incubation, 40 µL of PRP (plus or minus inhibitor) was quickly added to wells containing the lyophilised platelet agonists. PPP was added to four agonist-free wells to provide a signal equivalent to 100% aggregation. PRP was also added to agonist-free wells and wells containing vehicle alone as a 0% aggregation control. Plates were then placed on a thermal shaker (BioShake iQ, QInstruments, Jena, Germany) for 5 minutes (37°C, 1,200 revolutions per minute). Light absorbance was read at 595 nm in a 96-well plate reader (SPECTROstar Nano, BMG LABTECH, Aylesbury, United Kingdom).

Percentage aggregation was calculated using absorbance values for PPP (100%) and PRP (0%) in agonist-free wells as reference values. A visual inspection of concentration–response curves was conducted to remove any clearly erroneous curves (deviating from a sigmoid shape). Data were also removed if they showed no response, with response defined as greater than 30% aggregation in response to two doses of agonist.

### Vasodilator-Stimulated Phosphoprotein Phosphorylation

Vasodilator-stimulated phosphoprotein (VASP) is a platelet protein whose phosphorylation is modulated by cyclic adenosine monophosphate (cAMP). 17 cAMP production is inhibited by the action of G _αi_ on adenylate cyclase secondary to activation of P2Y _12_ receptors. 18 The degree of VASP phosphorylation can thus be used as a marker of P2Y _12_ activity. 19 The VASPFix assay 20 was used to quantify VASP phosphorylation.

Aliquots of PRP were incubated for 6 minutes in one of three conditions: phosphate-buffered saline (PBS), a prostacyclin analogue iloprost (1 nM), or ADP (5 µM) + iloprost (1 nM) and mixed in a 1:5 ratio with VASPFix (Platelet Solutions Ltd., Nottingham, United Kingdom), vortexed, and snap frozen on dry ice. Samples from high altitude were transported to the United Kingdom for analysis on dry ice by a specialist company.

Flow cytometry was performed and the median fluorescein isothiocyanate fluorescence (mf) recorded for each condition. Iloprost induces maximal VASP phosphorylation with PBS acting as a negative control.

If iloprost did not induce phosphorylation (i.e., if mf (iloprost) < mf (saline)), it was assumed that there was a technical failure and these samples removed from the analysis. Similarly, if mf (iloprost + ADP) was greater than mf (iloprost), the sample was also removed from analysis.

### Full Blood Count

Three millilitres of blood were collected into an ethylenediaminetetraacetic acid blood tube (Sarstedt Ltd., Leicester, United Kingdom) and samples analysed within 24 hours by clinical haematology laboratories (NHS Lothian Laboratories, Edinburgh, United Kingdom, and SELADIS, Universidad Mayor de San Andrés, La Paz, Bolivia).

### Statistics

Optimul aggregometry data were fit to Eq. (1) by least squares, non-linear regression using the scipy.optimize library (for Python 2.7.2).

Eq. (1) : Agonist-response equation where *y* is the percentage aggregation, *x* is log[agonist], EC _50_ the concentration resulting in half maximal aggregation, Top/Bottom the maximum/minimum aggregation and the Hill Slope the steepness of the curve. For studies examining the dose–response curve of cangrelor, IC _50_ replaces EC _50_ .



The concentration resulting in half-maximal aggregation (EC _50_ ) was used to compare curves from each time point. For comparisons between ADP concentration–response curves in the presence of a fixed antagonist, maximum response ( *R*_max_ ) to ADP was also calculated.

Outlier identification was based on difference of fits analysis. 21 The effect of each point on EC _50_ (ΔEC _50_ ) and Hill Slope (ΔHill) was calculated by iterative removal of points and refitting the curves. ΔEC _50_ and ΔHill values were reviewed for comparable data (all points in all concentration–response curves for one agonist at one time point) and any points falling out with two standard deviations (ΔEC _50_ ) or four standard deviations (ΔHill) were considered outliers. Additionally, for concentration–response curves in the presence of inhibitors, if the final point was less than 75% of the point preceding it, it too was considered an outlier, to ensure validity of the *R*_max_ parameter.

Wherever possible, paired statistics were used; however, the optimul aggregometry data were unpaired. For paired comparisons of two time points, paired Student's *t* -tests were performed using the scipy.stats library. 22 For paired data compared over three time points (SpO _2_ ), a one-way repeated-measure analysis of variance (ANOVA) was conducted with a Tukey's HSD post hoc test using Prism 5.0 (GraphPad, La Jolla, California, United States). For optimul aggregometry data, comparisons between three time points were analysed using one-way ANOVA with Tukey's HSD post hoc test using R 3.4.2. 23 Whenever multiple comparisons were made, between multiple agonists or conditions, *p* -values reported were adjusted using a Bonferroni correction. Statistical significance was set as 0.05.

## Results

Baseline characteristics of subjects are summarised in Table 1 .

**Table 1:** Baseline characteristics

Characteristic	APEX 5 cohort
Sex (male/female)	9/20
Mean BMI (range)	22.3 (17.6–28.8)
Mean age (range)	20.7 (18–26)

Note: Body mass index (BMI) has units kg/m ^2^ , and age is measured in years.

**Fig. 2:**
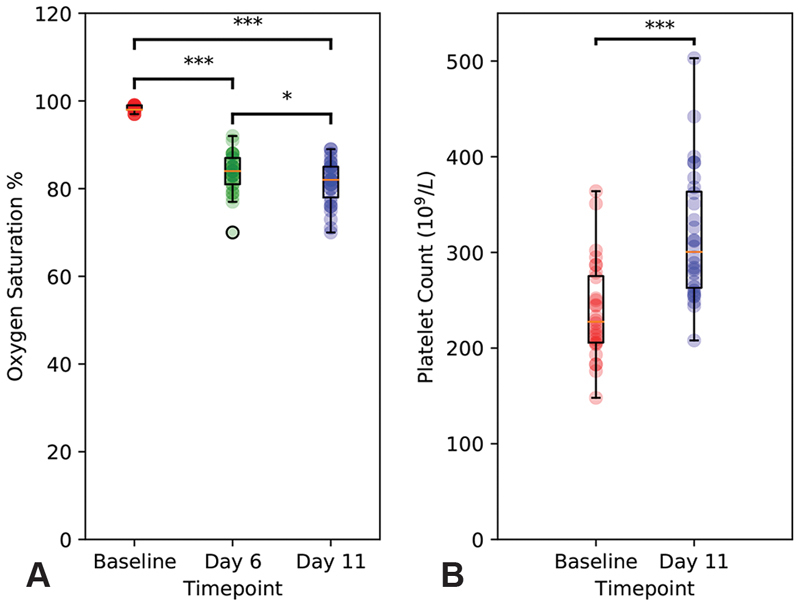
Platelet counts and oxygen saturations. ( **A** ) Oxygen saturation was measured at baseline and on day 6 and day 11 of the expedition. Data points are represented as semi-translucent circles, with summary box plots superimposed. Data were analysed by a one-way repeated-measure analysis of variance (ANOVA) followed by a Tukey's HSD test. ( **B** ) Platelet count was measured at baseline and on day 11 of the expedition. Data points are represented as semi-translucent circles, with summary box plots superimposed. Data were analysed by paired Student's *t* -test. *** *p* < 0.001, * *p* < 0.05.

All but one subject completed the study, and data have been included until dropout for unpaired analyses. The sojourn at high altitude induced a marked hypoxaemia ( Fig. 2A ), which was slightly more pronounced on day 11 than day 6. Platelet counts were also significantly elevated by hypoxia ( Fig. 2B ). Haemoglobin, haematocrit and white cell count data are provided in Supplementary Table S1 (available in the online version).

### Hypoxic Platelets are Less Sensitive to TRAP-6 Amide

**Fig. 3:**
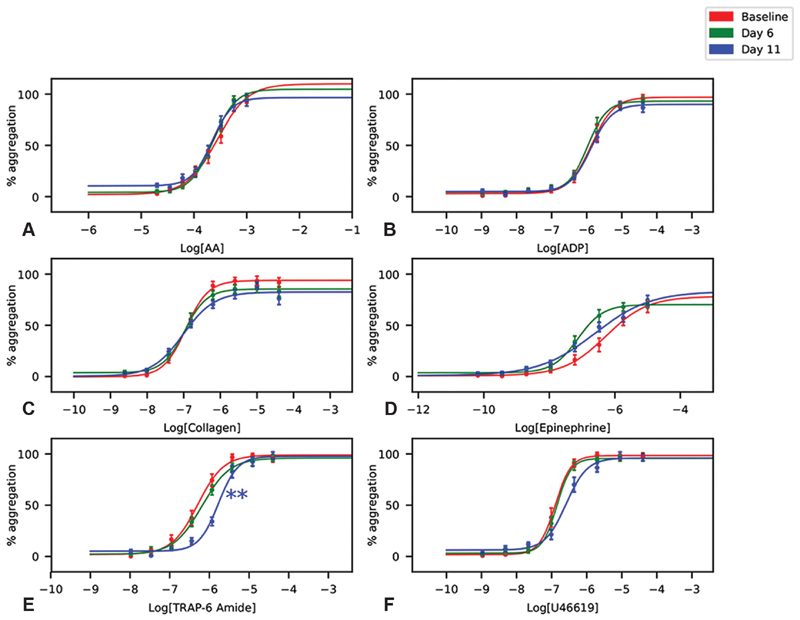
The effect of hypoxia on platelet activation pathways. Dose–response curves of platelet aggregation in response to ( **A** ) arachidonic acid (AA), ( **B** ) adenosine diphosphate (ADP), ( **C** ) collagen, ( **D** ) epinephrine, ( **E** ) thrombin receptor-activating peptide (TRAP)-6 amide and ( **F** ) U46619. Data are mean percentage aggregation ± standard error of the mean (SEM), and best fit curves optimised to these mean values. EC _50_ s were compared by one-way analysis of variance (ANOVA) followed by Tukey's HSD post hoc tests where appropriate. *p* -Values reported were adjusted to account for family-wise error rate using Bonferroni corrections. ** *p* < 0.01 vs. baseline.

The effect of hypoxia on various platelet agonists was compared between baseline, day 6 and day 11. No platelet activation pathways were found to be sensitised by hypoxia; however, platelets became less sensitive to TRAP-6 amide ( Fig. 3E , Table 2 , *p* < 0.01). This effect was only present on day 11.

**Table 2:** Summary results of optimul aggregometry data

EC _50_ values			
	Baseline	Day 6	Day 11
AA	–3.695 (0.053)	–3.696 (0.039)	–3.715 (0.064)
ADP	–5.990 (0.096)	–6.037 (0.122)	–5.912 (0.078)
Collagen	–6.776 (0.111)	–7.009 (0.107)	–6.853 (0.088)
Epinephrine	–6.339 (0.194)	–6.805 (0.144)	–6.584 (0.140)
TRAP-6 amide	–6.314 (0.105)	–6.221 (0.064)	–5.759 (0.084) a
U46619	–6.979 (0.122)	–6.652 (0.099)	–6.545 (0.122)
ADP + MRS2500	–5.280 (0.082)	–5.222 (0.063)	–5.244 (0.064)
ADP + Cangrelor	–5.186 (0.062)	–5.318 (0.068)	–5.335 (0.079)
***R***_**max**_ **values**			
ADP + MRS2500	95.230 (2.835)	100.413 (2.159)	96.638 (2.788)
ADP + Cangrelor	80.005 (4.177)	97.927 (2.557) a	97.186 (2.398) a

Abbreviations: AA, arachidonic acid; ADP, adenosine diphosphate; PRP, platelet-rich plasma; TRAP, thrombin receptor-activating peptide.

Note: Dose–response curves of platelet aggregation were performed in response to AA, ADP, collagen, epinephrine, TRAP-6 amide, and U46619. Aggregometry was also performed on PRP incubated for 30 minutes with 1 µM MRS2500 (P2Y _1_ inhibitor) or 100 nM cangrelor (P2Y _12_ inhibitor) with ADP as the agonist. For experiments with inhibitors, the maximal response ( *R*_max_ ) was also calculated. EC _50_ and *R*_max_ values were compared by one-way analysis of variance (ANOVA) followed by Tukey's HSD post hoc tests where appropriate. *p* -Values reported were adjusted to account for family-wise error rate using Bonferroni corrections.

a *p* < 0.01 vs. baseline.

### Hypoxia Modulates Purinergic Signalling

**Fig. 4:**
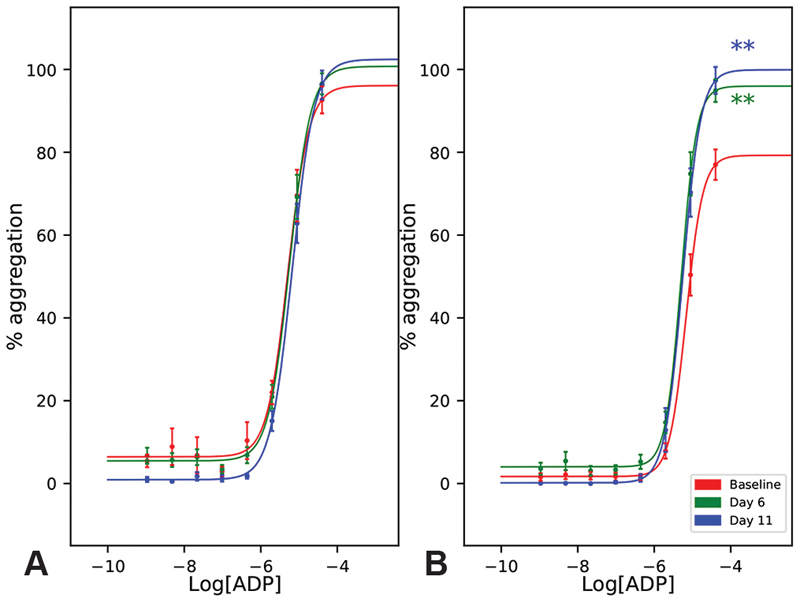
The effect of hypoxia on adenosine diphosphate (ADP)-induced platelet aggregation in the presence of fixed doses of inhibitors. Platelet-rich plasma (PRP) was incubated for 30 minutes with ( **A** ) 1 µM MRS2500 (P2Y _1_ inhibitor) or ( **B** ) 100 nM cangrelor (P2Y _12_ inhibitor). Data are mean percentage aggregation ± standard error of the mean (SEM), and best fit curves optimised to these mean values. EC _50_ and *R*_max_ values were compared by one-way analysis of variance (ANOVA) followed by Tukey's HSD post hoc tests where appropriate. *p* -Values reported were adjusted to account for family-wise error rate using Bonferroni corrections. ** *p* < 0.01 vs. baseline.

In the presence of 1 µM P2Y _1_ inhibitor MRS2500, both EC _50_ and *R*_max_ were unchanged by hypoxia ( Fig. 4A , Table 2 ). Likewise, in the presence of 100 nM P2Y _12_ inhibitor cangrelor, EC _50_ remained unchanged after 11 days of hypoxia. While EC _50_ remained unchanged at altitude, *R*_max_ was markedly increased on both days 6 and 11 compared with baseline ( Fig. 4B , Table 2 , *p* < 0.01).

### Hypoxia Modulates Basal VASP Phosphorylation in Platelets

**Fig. 5:**
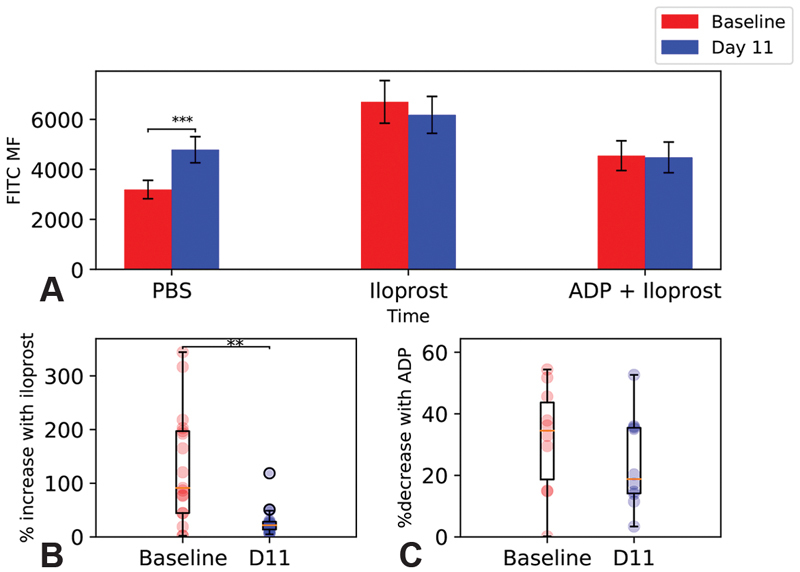
The effect of hypoxia on platelet vasodilator-stimulated phosphoprotein (VASP) phosphorylation. Platelet-rich plasma (PRP) was incubated for 6 minutes with phosphate-buffered saline (PBS), iloprost or adenosine diphosphate (ADP) + Iloprost before addition of VASPFix. Flow cytometry was used to identify the fluorescein isothiocyanate (FITC) median fluorescence (MF), reflecting the degree of VASP phosphorylation. ( **A** ) Raw FITC MF for each condition at each time point. Data are mean FITC MF ± standard error of the mean (SEM). Data were compared by paired *t* -tests with *p* -values adjusted by Bonferroni correction. ( **B** ) Percentage increase in FITC MF induced by addition of iloprost. ( **C** ) Percentage decrease induced by ADP addition. ( **B** , **C** ) Individual data points are represented by semi-translucent circles with box plots superimposed. Data were compared by paired *t* -tests. ** *p* < 0.01, *** *p* < 0.001.

Compared with baseline, after 11 days at high altitude there was a highly significant increased mf in the PBS-treated samples ( Fig. 5A ). There were no differences between baseline and day 11 in other conditions ( Fig. 5A ). There was a marked reduction in the mf percentage increase induced by iloprost at high altitude ( Fig. 5B ), but no change in mf reduction induced by the addition of ADP ( Fig. 5C ).

## Discussion

There is a growing body of evidence that hypoxia induces hyper-reactivity in platelets, a phenomenon important to both altitude physiology and sea-level pathophysiology. We found that hypoxia increases maximal aggregation to ADP in the presence of cangrelor and down-regulates platelet sensitivity to TRAP-6 amide. However, we did not demonstrate sensitisation to any of the platelet activators tested. Finally, and more strikingly, we have demonstrated modulation of basal VASP phosphorylation, a key step in the P2Y _12_ signalling pathway.

Our results contrast with a previous study from our group which reported increased platelet sensitivity to ADP. 6 Our previous findings were based on Multiplate analysis and discrepancies could be related to the impact of platelet count on the two different assays: Multiplate is more sensitive to platelet count than optimul aggregometry. 24 25 This, however, would not explain why our previous findings were specific to ADP. Perhaps more likely is that differences relate to the parameter of platelet function that was tested. Multiplate is based on electrical-impedance aggregometry measured in real-time over 6 minutes, with the area under the impedance time curve as the final outcome. 26 This outcome is therefore dependent on both the final amplitude of the response and the rate at which this is reached. Optimul aggregometry, however, is based on one measurement at 5 minutes. Conceivably, differences in the ADP sensitivity could be time dependent, given the time sensitivity of phosphorylation 27 and changes in purinergic receptor expression 28 induced by ADP stimulation.

While we have not resolved the uncertainty surrounding ADP sensitivity, our study did reveal interesting changes in purinergic signalling. The *R*_max_ induced by ADP in the presence of 100 nM cangrelor was significantly higher at altitude ( Fig. 4B , Table 2 ). This could be due to increased P2Y _1_ receptor activity. An increase in P2Y _1_ activity may imply a difference in receptor expression or modulation of receptor activity since G-protein-coupled receptor activity can be regulated by multiple mechanisms. 29 Increased P2Y _1_ activity would provide an interesting link to the proteomic data of Tyagi et al who described increased calpain expression and activity in rats exposed to hypoxia for 6 hours, 12 since a downstream consequence of P2Y _1_ activation is increased intracellular calcium. 30

Well-controlled, high-altitude expeditions are excellent models of acute hypoxia 31 and their findings are often relevant to hypoxic patients at sea level. In this regard, patients with chronic hypoxic diseases such as chronic obstructive pulmonary disease and obstructive sleep apnoea (OSA) have an increased risk of thrombotic events such as myocardial infarction and stroke. 32 33 Furthermore, these conditions are also associated with increased platelet reactivity, which in the case of OSA is reversed on correction of hypoxia. 34 35 36 However, the clinical relevance of isolated, increased P2Y _1_ activity is unclear since overall sensitivity to ADP was unaltered ( Fig. 3B , Table 2 ). Nonetheless, increased P2Y _1_ receptor activity has been postulated to contribute to P2Y _12_ inhibitor resistance, 37 38 although evidence for this is lacking and to our knowledge no study has investigated the impact of hypoxia on P2Y _12_ inhibitor resistance.

**Fig. 6:**
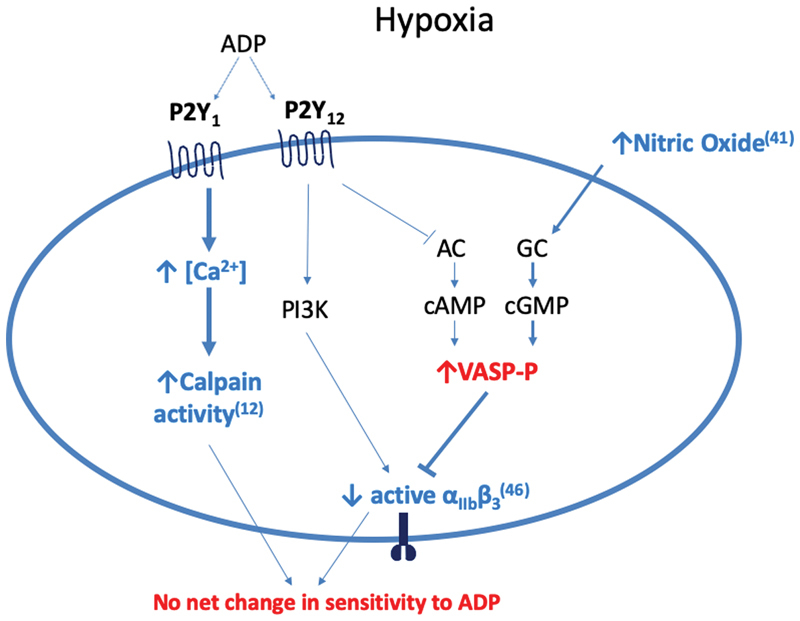
Proposed model of hypoxia-induced changes to purinergic signalling. Our findings are in red, while those of other studies examining the impact of hypoxia on platelet signalling are in blue. Our data suggest that hypoxia up-regulates the basal level of vasodilator-stimulated phosphoprotein (VASP) phosphorylation, a key determinant of platelet aggregation downstream of the P2Y _12_ receptor. Other work demonstrating increased nitric oxide (NO) levels at altitude provides a possible mechanism for this finding. 41 Phosphorylated-VASP (VASP-P) inhibits the expression of active α _IIb_ β _3_ expression, which links to Kiouptsi et al's finding that hypoxic platelets have lower expression of active α _IIb_ β _3_ in response to adenosine diphosphate (ADP). 46 Increased P2Y _1_ pathway activity would be consistent with Tyagi et al's finding that calpains are up-regulated by hypoxia. 12 Since we found no change in overall sensitivity to ADP, it may be that the changes produced by hypoxia in these two pathways counteract one another. AC, adenylate cyclase; ADP, adenosine diphosphate; cAMP, cyclic adenosine monophosphate; cGMP, cyclic guanine monophosphate; GC, guanylate cyclase; PI3K, phosphoinositide 3-kinase.

An alternative explanation for the change in *R*_max_ with cangrelor is an increase in P2Y _12_ expression at altitude. Interestingly, a recent whole blood microarray study showed a twofold increase in P2Y _12_ expression in well acclimatised sojourners at high altitude. 39 These findings were not validated in isolated platelets or by quantitative polymerase chain reaction. With either an increase in P2Y _1_ or P2Y _12_ expression, a change in sensitivity to ADP might be expected in the absence of inhibitors. However, as we detected no change in sensitivity to ADP, we propose that our finding of increased basal VASP phosphorylation is a compensatory mechanism that is also activated by hypoxia. Together with a lack of change in maximum phosphorylation induced by iloprost ( Fig. 5B ) and a reduction in the percentage increase in phosphorylation induced by iloprost ( Fig. 5C ), this suggests that the basal ratio of VASP:VASP-P is altered by hypoxia. Although we did not observe reduced P2Y _12_ activity in response to ADP ( Fig. 5A, C ), we only studied a single high concentration of ADP (5 µM), a concentration which induces near-maximal aggregation ( Fig. 3B ). It may be that lower concentrations of ADP are unable to overcome increased basal VASP phosphorylation. If this is the case, it could represent a compensatory response to increased P2Y _1_ receptor activity or P2Y _12_ expression, the explanations proposed for the reduction in cangrelor efficacy. VASP-P is regulated by several mediators that are altered in hypoxic conditions. For example, exhaled nitric oxide (NO) levels increased over 48 hours in healthy subjects exposed acutely to 4,559 m, 40 while raised circulating cyclic guanosine monophosphate (cGMP) levels and NO metabolites (nitrite and nitrate) were reported following gradual ascent to 5,200 m. 41 NO inhibits platelet aggregation, at least in part via cGMP-mediated VASP phosphorylation as platelet adhesion to injured vessel walls could not be inhibited by NO in VASP-deficient mice. 42 Interestingly, recent work demonstrated that nitrite led to VASP phosphorylation in isolated platelets in the presence of deoxygenated red blood cells. 43 Thus, either increased NO synthase or nitrite reductase activity could explain our finding of increased basal VASP phosphorylation at altitude. 44 Fig. 6 summarises the proposed changes to purinergic signalling induced by hypoxia; however, this model will require further investigation to confirm its validity.

Kiouptsi et al examined the effects of brief exposure (30 minutes) of washed human platelets to extreme and moderate hypoxia (1 and 8% oxygen, respectively) on ex vivo expression of activated α _IIb_ β _3_ (a key integrin in platelet aggregation 45 ) and aggregation. They reported that platelets exposed to extreme hypoxia had reduced expression of activated α _IIb_ β _3_ in response to ADP stimulation. 46 Our proposed model of hypoxia-induced alterations to purinergic signalling may offer a mechanistic explanation to these findings since an increase in VASP phosphorylation would attenuate P2Y _12_ activity and reduce ADP-induced activation of α _IIb_ β _3_ . 47 48 49 Kiouptsi et al also demonstrated that brief, extreme hypoxic exposure (1% oxygen, 30 minutes) reduced aggregation of hypoxic washed platelets in response to TRAP-6. However, sensitivity of PRP to TRAP-6 amide was unchanged. 46 Our data did not show any difference in TRAP-6 sensitivity on day 6 ( Fig. 3E ), but reduced sensitivity was observed after a longer duration of hypoxia. It could be that hypoxia-induced changes in sensitivity to TRAP-6 amide evolve over time, and only after 11 days was the effect on platelets pronounced enough to be seen in PRP. Interestingly, transcription of the receptor for TRAP-6 amide, protease-activated receptor 1, has also previously been shown to be down-regulated in hypoxic cancer cells, 50 but this has not been investigated in platelets and would be an interesting topic for further research.

This study has several limitations. First, it is difficult to predict the clinical relevance of subtle changes to signalling when overall sensitivity to the agonist is not altered. Additionally, our study only examined the purinergic pathways in detail, and it is possible that other subtle differences exist in other signalling pathways. Finally, our study had a slight gender imbalance, and sex has been shown to affect platelet aggregation and purinergic signalling, 51 implying our results may be more relevant to women than men.

Detailed investigation of purinergic signalling provided evidence of increased VASP phosphorylation and impaired inhibition of aggregation by a P2Y _12_ antagonist. We did not, however, find evidence of increased sensitivity to any platelet agonists despite the substantial evidence of hypoxia-induced platelet hyper-reactivity in the literature. However, the observed changes in aggregation in response to an ADP antagonist are of potential therapeutic significance to high-altitude sojourners and hypoxic sea-level patients prescribed platelet inhibitors and warrant further investigation.

## Notes

**What is known about this topic?**

- Exposure to hypobaric hypoxia is thought to induce a thrombogenic phenotype, with increased risk of stroke in those residing at high altitude.
- The platelet proteome is altered by hypoxia and previous work has suggested platelet sensitivity to ADP is increased at altitude.

**What does this paper add?**

- Using optimul aggregometry, we show no significant changes in the dose–response of several platelet receptor agonists.
- Hypoxia suppressed the extent to which cangrelor inhibited platelet aggregation to ADP, a finding with potential implications for hypoxic patients taking platelet inhibitors.
