# Supplementary material for: Hypoxia Modulates Platelet Purinergic Signalling Pathways
Source: Thromb Haemost. 2019 Dec 13;120(2):253–61. doi: 10.1055/s-0039-3400305 (PMC7286126; doi:10.1055/s-0039-3400305)
Supplement: Supplementary file 1 — Supplementary Material [file 10-1055-s-0039-3400305-s190243.pdf]

Supplementary Table S1 Full blood count (FBC) analysis

	Baseline	Day 11		*p*-Value
Haematocrit	0.40 ± 0.006	0.46	± 0.007	< 0.0001
Haemoglobin (g/L)	137.6 ± 2.42	150.7	± 2.74	< 0.0001
Platelets ($\times 10^9$/L)	240 ± 9.83	317	± 12.9	< 0.0001
White blood cells ($\times 10^9$/L)	5.9 ± 0.29	7.4	± 1.78	0.002

Note: Blood was taken into tubes containing ethylenediaminetetraacetic acid (EDTA) and FBC tests were performed in commercial laboratories at baseline and on day 11 at 4,700 m. Data are mean with standard error of the mean (SEM) and differences between baseline and Day 11 were analysed by paired Student's *t*-tests.
